# Latest results from the dose‐expansion part (Part 2) of the Brainshuttle™ AD study of trontinemab in people with Alzheimer’s disease

**DOI:** 10.1002/alz70859_104293

**Published:** 2025-12-25

**Authors:** Luka Kulic, Fabien Alcaraz, Gregory Klein, Stephen Salloway, Carsten Hofmann, João A. Abrantes, Stella Yilmaz, Denise Sickert, Maddalena Marchesi, Jakub Wojtowicz, Andres Schneider, Ruth Croney, David Agnew, Silke Ahlers, Paul Delmar, Hanno Svoboda, Iris Wiesel

**Affiliations:** ^1^ F. Hoffmann‐La Roche Ltd, Basel, Basel Switzerland; ^2^ F. Hoffmann‐La Roche Ltd, Basel Switzerland; ^3^ Butler Hospital and Warren Alpert Medical School, Brown University, Providence, RI USA; ^4^ Roche Products Ltd, Welwyn Garden City United Kingdom; ^5^ Excelya Germany GmbH, Mannheim Germany

## Abstract

**Background:**

The anti‐amyloid Brainshuttle™ antibody trontinemab is currently being investigated in the Phase Ib/IIa Brainshuttle™ AD study in people with mild cognitive impairment due to Alzheimer’s disease (AD) or mild‐to‐moderate AD (NCT04639050). The objective of this presentation is to share the latest safety, pharmacokinetics, and pharmacodynamics (PD) results from the ongoing dose‐expansion part (Part 2) of the study.

**Method:**

In the initial 28‐week dose‐escalation part (Part 1) of the Brainshuttle™ AD study, 60 participants were randomized to four sequential dose cohorts: 0.2 mg/kg (Cohort 1), 0.6 mg/kg (Cohort 2), 1.8 mg/kg (Cohort 3), and 3.6 mg/kg (Cohort 4). Following review of emerging data, the two higher dose levels (Cohort 3 and Cohort 4) that showed favorable PD and safety results in Part 1 were investigated further in the dose‐expansion part (Part 2) of the study (+60 participants per expanded‐dose cohort; 28 weeks of treatment; 4:1 active:placebo randomization ratio, identical to Part 1).

**Result:**

A recent analysis of the Part 1 results revealed dose‐dependent amyloid plaque lowering on amyloid positron emission tomography (PET) imaging at all active dose levels (data snapshot: Sep 2, 2024). At the two higher dose levels (1.8 and 3.6 mg/kg), a very rapid amyloid plaque depletion was observed, and most of those participants with available PET scans (67–75%) became amyloid negative (≤24 centiloids) after 28 weeks of treatment. These rapid and robust effects on amyloid PET were accompanied by early and pronounced effects on several key downstream biomarkers in cerebrospinal fluid. The incidence of amyloid‐related imaging abnormalities (ARIA) was low (one ARIA – edema and one ARIA – hemosiderosis occurred in Part 1 [n=60] as of the data snapshot on Sep 2, 2024). Detailed safety and PD (amyloid PET) results from the completed Cohorts 3 and 4 in Part 2 will be presented from the most recent available data snapshot.

**Conclusion:**

Results from the completed Cohorts 1–4 in Part 1 of the Brainshuttle™ AD study suggest that early and pronounced effects on several key markers of AD pathophysiology can be achieved with trontinemab treatment with very limited incidence of ARIA.

